# A comprehensive strategy for the analysis of acoustic compressibility and optical deformability on single cells

**DOI:** 10.1038/srep23946

**Published:** 2016-04-04

**Authors:** Tie Yang, Francesca Bragheri, Giovanni Nava, Ilaria Chiodi, Chiara Mondello, Roberto Osellame, Kirstine Berg-Sørensen, Ilaria Cristiani, Paolo Minzioni

**Affiliations:** 1 Department of Electrical, Computer, and Biomedical Engineering, Università di Pavia, Via Ferrata 5A, 27100, Pavia, Italy; 2 Institute of Photonics and Nanotechnology, CNR & Department of Physics, Politecnico di Milano, Piazza, Leonardo da Vinci 32, 20133 Milano, Italy; 3 Department of Biomedical Science and Translational Medicine, Università di Milano, Via F.lli Cervi 91, 20090 Segrate, Italy; 4 Institute of Molecular Genetics (IGM), CNR, Via Abbiategrasso 207, 27100 Pavia, Italy; 5 Department of Physics, Technical University of Denmark, DK-2800 Kgs, Lyngby, Denmark

## Abstract

We realized an integrated microfluidic chip that allows measuring both optical deformability and acoustic compressibility on single cells, by optical stretching and acoustophoresis experiments respectively. Additionally, we propose a measurement protocol that allows evaluating the experimental apparatus parameters before performing the cell-characterization experiments, including a non-destructive method to characterize the optical force distribution inside the microchannel. The chip was used to study important cell-mechanics parameters in two human breast cancer cell lines, MCF7 and MDA-MB231. Results indicate that MDA-MB231 has both higher acoustic compressibility and higher optical deformability than MCF7, but statistical analysis shows that optical deformability and acoustic compressibility are not correlated parameters. This result suggests the possibility to use them to analyze the response of different cellular structures. We also demonstrate that it is possible to perform both measurements on a single cell, and that the order of the two experiments does not affect the retrieved values.

During the last decade, the rapid development of microfluidic circuits and lab-on-chip devices for cell studies opened new interesting perspectives for cellular biology, in particular regarding the possibility to analyze the biophysics and biomechanics of single cells^1^,^2^,^3^,^4^. Carcinogenesis is one important biological field in which such lab-on-chip devices can play a relevant role. Several studies demonstrated that cellular neoplastic and malignant transformation are closely connected with significant changes in the cytoskeleton, which are in turn related to changes in the mechanical properties of the cell^5^,^6^,^7^. Thus, since the mechanical properties of cells seem to be directly associated with the cellular status^8^,^9^,^10^, the possibility to use them as label-free sensitive markers (e.g. to distinguish cancer cells from healthy ones), to differentiate specific cells within a heterogeneous population, or even to perform other mechanical-based functionalities (like heterotypic cell pairing^11^,^12^), appears as a promising way for innovative biological studies.

At the state of the art, many different methods and techniques were proposed to measure cellular mechanical properties either quantitatively or qualitatively. To give a few examples, in the atomic force microscopy technique the cantilever tip is attached to the cells’ surface and the relative indentation depth at constant force is used to determine the cellular Young’s modulus^13^,^14^ or to study cell plasma membrane tension^15^; micropipette aspiration applies a negative pressure in the micropipette to form a gentle suction on the cell and study the local membrane deformation at the contact area^16^,^17^; optical tweezers or magnetic tweezers with microbeads attached to the cell membrane can apply a very large force to the cell surface and allow for the measurement of cellular viscoelastic moduli^18^,^19^; microfluidic constriction channels for cell migratory capability analysis allow studying both active and passive cell mechanical properties^20^,^21^,^22^,^23^. However, most of these methods require a direct cell-device contact, which could damage the studied cells during the measurement, or some of them only probe a small part of the whole cell, providing a partial data recovery and analysis. Furthermore, these techniques often require quite complicated experimental preparations and then offer a relatively limited throughput. In contrast, techniques based on purely hydrodynamic cell stretching^24^ can offer a significant increase of the throughput, but do not allow for single cell studies or even to reuse the analyzed cells, two features that are possible and even inherent when using optical trapping for sorting based on mechanical characteristics^25^,^26^.

The optical stretcher^27^ has been widely and successfully applied for many different cell studies. Different from optical tweezers^28^,^29^, it exploits optical forces to induce cell, or small organelle, deformation^7^,^30^ and it can be easily integrated inside a microfluidic device^31^,^32^,^33^, which makes it an efficient and contactless tool to investigate cellular mechanical properties at the single cell level. Several papers already proved that cell optical deformability allows distinguishing healthy, tumorigenic and metastatic cells, and also showed that optical stretching can be used to reveal the effects of drug treatments on the mechanical response of the cell^5^,^17^,^22^,^34^. Additionally, a series of recent papers exploits the optical stretcher as a tool to study the effect of temperature on cell mechanics to better understand cellular thermorheology^35^,^36^,^37^,^38^.

Acoustofluidics, the combination of acoustics and microfluidics, has also been used increasingly during the last five years. It utilizes ultrasonic standing wave forces and acoustic streaming^39^ inside the microfluidic system for microparticle and cell manipulation and separation^40^,^41^,^42^,^43^. Acoustofluidics benefits from acoustic forces allowing for rapid actuation, programmable capability, simple operation and high throughput^44^. Similarly to the optical stretcher, it can provide a contactless way for cell analysis and can also be easily integrated within a lab-on-chip device. Based on this technique, some studies on mechanical properties of cells in terms of their acoustic compressibility already demonstrated that cancer cells generally have a higher compressibility than their normal counterparts^45^,^46^,^47^.

At present, however, a complete procedure that allows for reliable compressibility measurements, based on a full on-chip characterization of all the relevant parameters, has not been reported in the literature. In this work we exploit a microfluidic setup, which combines optical and acoustic actuators, to perform cell mechanics characterization in terms of both cellular optical deformability (OD) and acoustic compressibility (AC). The precise determination of all the system parameters required for a proper estimation of cellular OD and AC (i.e. cell culture-medium refractive index, viscosity and density, and cell size) is achieved by a series of specifically designed experiments. Among them, we report a new method to evaluate, in a non-destructive manner, the size of the optical beam, emitted by an integrated waveguide, inside a microchannel. As a final result, we measure the OD and AC of two human cancer cell populations characterized by different metastatic potential and we compare these two parameters. It is important to highlight that this technique allows obtaining different mechanical measurements on a single cell, and the low measurement throughput is compensated by the possibility to perform single-cell selection and recovery.

## Results

### System parameters assessment

A fundamental step to obtain a reliable estimation of the OD and AC of a cell is the precise knowledge of the system parameters involved; for this reason, before discussing measurement results on cells, we report the characterization of the cell buffer and of the optical beams.

#### Refractive index and density of the cell buffer

**Figure 1:**
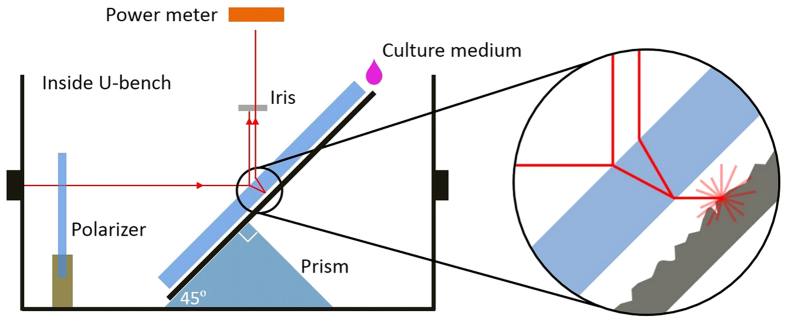
Local refractive index measurement setup. The incidence angle of laser beam on the plate is fixed at 45° because of the mounting prism.

The refractive index and the density of the cell buffer, the cell culture medium in our experiments, are two critical parameters for optical forces and AC evaluation, which can also vary due to the different compositions and preparation methods. In order to assess the refractive index of the culture medium at the wavelength of interest for our experiments (1070 nm, in the near-infrared range) we realized a simple setup, shown in Fig. 1. The whole configuration is located inside an optical “U-bench” so as to have an easy access of the laser beam. A 5 mm thick BK7 optical plate is used to spatially offset the beams reflected by the two interfaces (air-glass and glass-air), so as to avoid any interference between them. Moreover, by using a variable iris it was also possible to select one of the two reflected beams at a time. The optical slide was positioned on a dark and highly diffusive surface, with a small gap of about 1 mm, whose purpose is to remove additional reflections and hence to simplify the reflection coefficient calculation. The gap, initially filled by air (n_air_ = 1.000), was subsequently filled by cell buffer (n_buffer_), thus changing the amount of power reflected at the second interface (see Fig. 1). The ratio between the optical power reflected first at the glass-air interface (*P_2,a_*) and, after filling the space with cell buffer, at the glass-buffer interface (*P_2,b_*) is calculated by Eq. (1). Once the power ratio is known, it is thus possible to derive n_buffer_ exploiting the Fresnel equations and Snell’s law (the incidence angle on the plate is fixed at 45°). In addition, thanks to a free-space polarizer both the *σ* and *π* reflection coefficients can be separately assessed. By calculating the ratio of the power of the two beams, and thanks to the known value of the refractive index of BK7 (1.5066^48^ @1070 nm), the reflection coefficient at the glass-buffer interface can be immediately calculated.


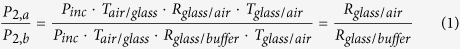


The obtained results, for both *σ*-polarized and *π*-polarized laser light, yielded a value of the culture medium refractive index of 1.327 ± 0.004. The culture medium density was determined by measuring on a high-precision scale the weight of a precisely measured volume of culture medium, and was obtained to be equal to 1050 ± 5 kg/m^3^.

#### Waveguide characterization and viscosity derivation

An interesting feature of lab-on-chip devices is the possibility to integrate optical functionalities, by realizing/including optical waveguides directly on-chip. In this case, instead of measuring the relevant beam parameters “in the waveguide”, or when the beam propagates out of the chip external surface, it would be desirable to characterize them directly inside the microchip, since possible fabrication imperfections or sidewalls roughness could affect the beam properties. For this reason, we implemented a simple method, based on optical shooting experiments^49^, for non-destructive evaluation of the beam properties inside the microchannel.

**Figure 2:**
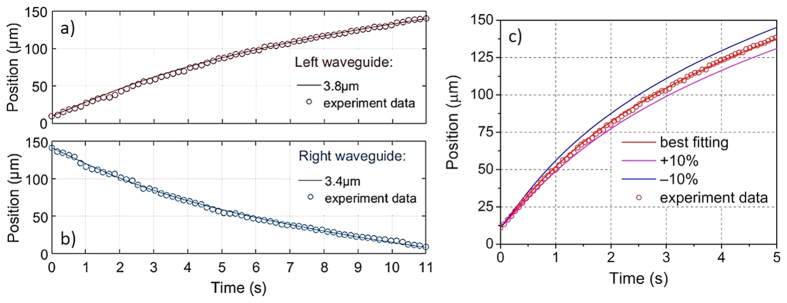
Microbead trajectory from optical shooting measurements: experimental data (circles) are shown together with the fitting curves (theoretically predicted). (**a**,**b**) Show the trajectories of a polystyrene bead suspended in pure water with the best fitting by considering different beam waist values: 3.8 and 3.4 μm for the left and right beam respectively. (**c**) Shows the trajectory of the same bead but immersed in cell culture medium, together with the fitting curves obtained considering the retrieved viscosity parameter (0.78 mPa. s) and a possible 10% viscosity deviation, to demonstrate the importance of a correct viscosity evaluation. For sake of clarity, the data reported in each chart are obtained by down-sampling the real data. Error bar (not displayed) would be smaller than circle diameter. Position is measured starting from the channel’s left border (0 μm) to the right one (150 μm).

A suspension of monodispersed polystyrene beads in pure water, with a diameter of 7.3 μm, is introduced in the chip and a single microbead is initially trapped. The trapping position is then progressively shifted across the microchannel, by reducing the optical power in one waveguide (as explained in the materials and methods section, this is achieved by putting a variable optical attenuator inside one of the fiber-to-fiber U-benches). Once the bead reaches the microchannel sidewall, the stronger optical beam is abruptly blocked and optical shooting of the microbead by the only active beam is obtained and recorded. From the analysis of the recorded video, the curve of the microbead position as a function of time can be extracted, as shown in Fig. 2(a,b).

In order to evaluate the optical force applied on the microbeads, Gaussian beams were assumed to be freely-propagating in the microchannel and the standard paraxial ray-optics decomposition was used to calculate the optical force produced on the beads. By comparing the experimentally retrieved curves of bead position as a function of time with those calculated by considering beams with different waist and optical power, it was possible to obtain both the beam waist, and the value of the optical power associated to each beam propagating inside the chip. Detailed information regarding the impact of the viscous drag force, the fitting procedure, and the used paraxial ray-optics decomposition can be found in literature^49^.

Figure 2(a,b) show the best fitting curves for both the left and right waveguides. The resulting waist values (3.8 and 3.4 μm for left and right beam respectively) are in good agreement with that obtained by analyzing the beam output (in air). It is interesting to notice that a small, but not negligible, difference between the beam waist values, obtained for the two waveguides, was observed. A possible explanation of this difference is that the non-ideal quality of the microchannel sidewalls (realized by chemical etching) induces a small scattering, which could be numerically simulated as a narrower beam waist and an increased beam divergence. It is important to highlight that our procedure allows for high precision in the determination of the optical force along the beam axis, which is the relevant parameter, even if fabrication imperfections may cause a non-perfect Gaussian intensity distribution of the beam. Additionally, the presented technique may also be used to estimate the overall losses from both fiber-to-waveguide coupling and propagation along the waveguide, by simply comparing the optical power value measured “in the U-bench” with those estimated, by the fitting procedure “in the channel”.

Once the force-profile produced by each beam propagating in the culture medium is calculated, a similar experiment can be exploited to evaluate the medium viscosity, by simply using it in place of water as the microbead suspension buffer. The viscosity of the medium in which the cells are suspended drastically impacts the drag force for any kind of phoresis-study, in particular the acoustophoresis trajectory analysis, that will be detailed in the following section. Taking into account the refractive index of the medium and the beam waist just assessed, the optical force can be calculated by paraxial ray-optics, and the microbeads displacement curves can now be fitted with the medium viscosity as the only free parameter. Using the previously reported data, we obtain a medium viscosity of 0.78 mPa s. It is interesting to notice that even a moderate variation of the viscosity parameter (e.g. ±10%) would significantly affect the calculated trajectories, as shown for reference in Fig. 2(c), where we report the experimental data (circles) with the best-fit curve and two additional curves in which we have increased and decreased the viscosity by 10% relative to the best-fitting viscosity value. For this reason, it is extremely important to independently measure the viscosity of exactly the same medium used for cell analysis under the same experimental conditions in order to obtain reliable cellular compressibility values. As a reference, the viscosity of water at 20 °C decreases by 2% when the temperature is increased by 1 °C.

### Cell measurements

Once the system parameters were fully characterized, we passed to analyze cell properties. First, we separately analyzed cellular AC, by acoustophoresis, and OD, by optical-stretching, of two human breast cancer cell line: MCF7 and MDA-MB231 (MDA in the following text); then we performed combined measurements to determine AC and OD of the same cell in order to compare and correlate these two parameters.

#### Cellular acoustophoresis and acoustic compressibility

Single cell AC measurements were carried out using the following procedure. After a single cell is trapped by the optical forces, the trapping position is shifted, as described in the materials and methods section. When the trapped cell has almost reached the microchannel sidewall, the laser source is completely switched off, so as to remove the optical force acting on the cell, and in the meantime the function generator is turned on to excite the piezo transducer. The cell movement is thus now completely determined by the acoustophoresis, and this movement is recorded by the CCD camera so that it is possible to extract the time-position curve and to determine, by a best-fitting procedure, the cell’s AC^45^,^50^.

**Figure 3:**
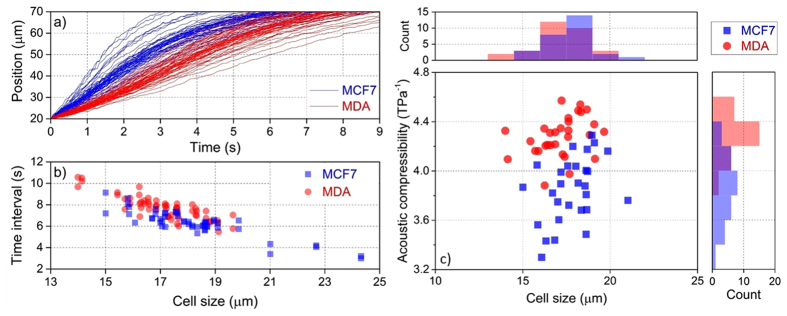
Cellular acoustophoresis trajectory and acoustic compressibility. (**a**) Acoustophoretic trajectories in common transverse movement from 20–70 μm for both MCF7 and MDA cells. (**b**) Time interval required for the transverse movement in (**a**) versus cell size. (**c**) Cellular acoustic compressibility versus cell size: MCF7 and MDA show a very similar cell size, 17.3 ± 1.0 μm, but different acoustic compressibility, 3.8 ± 0.3 TPa^−1^ for MCF7 and 4.3 ± 0.2 TPa^−1^ for MDA.

During each acustophoresis experiment, particular attention was paid to avoid any change in the efficiency of acoustic wave coupling from the piezo-actuator to the microchip. This was achieved by only perfusing the chip with pure culture medium when the cell sample to be analysed was changed, thus having the cell suspension and the buffer vials as the only part of the experimental apparatus that were mechanically touched during the procedure. It should be noted that the “starting position” of the cell in each measurement is not particularly relevant for the compressibility analysis, as the cell speed in each point of the microchannel is simply related to the equilibrium condition between the acoustophoretic force and the drag force. This detail, due to the fact that inertial effects can be neglected because of the extremely low (<10^−3^) Reynolds number, simplifies the analysis, as it is possible to consider the cell movement from a well-defined “starting line” to the node position of the channel center, even if the cell was initially in a slightly different position. In our case, by positioning the “starting line” at 20 μm from the sidewall and by measuring 60 cells (29 MCF7 and 31 MDA), we obtain the time-position trajectories shown in Fig. 3(a). In order to better highlight the impact of the cell size on the speed of cell movement, in Fig. 3(b), we also show the time intervals required by different cells to go from the “starting line” (@ 20 μm) to an ideal “finish line” (@ 70 μm from the sidewall).

As the studied cell samples and microbeads are significantly larger than 2 μm, the acoustic radiation force dominates relative to the streaming drag force^39^. For a simple 1D standing wave situation, this acoustic radiation force (*F*_*ac*_) can be described analytically by Eq. (2), where Φ represents the “acoustophoretic contrast factor”, *x* the transverse position of the bead, *R* the radius of the bead or cell, *k* the acoustic wave number, *E*_*ac*_ the position independent acoustic energy density, *ρ*_*cell*_ and *ρ*_*buffer*_ the densities of the cell and cell buffer medium, respectively and *β*_*cell*_ and *β*_*buffe*_ the compressibility values of cell and cell buffer medium, respectively.


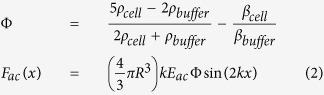


In Fig. 3(a,b) it is clearly shown that MCF7 cells are characterized by a faster movement across the microchannel than MDA cells and, by analyzing the bottom panel, it appears that larger cells have a shorter “transit-time” between the two considered lines. We explain this by noting that the acoustic force is proportional to the cell volume and thus larger cells are affected by a stronger acoustic force. Since the opposing drag depends on radius to the first power only, larger cells then move faster. It is therefore reasonable that the time interval required to travel a given distance will decrease with increasing cell size as shown in Fig. 3(b). Figure 3(b) also allows a preliminary evaluation of the cell compressibility: since the buffer fluid, the external driving voltage and the frequency of the piezo-actuator are unchanged, the behavior of cells within the same size range can be immediately compared: for every given cell size, MDA (red) dots tend to lay above the MCF7 (blue) ones, thus suggesting that the two cell lines have different compressibility.

To correctly estimate the cell compressibility value, we performed acoustophoresis-trajectory fitting^45^,^50^ by using Eq. (3), considering that during cell movement, the acoustophoretic force is balanced by the drag force produced by the cell culture-medium viscosity:


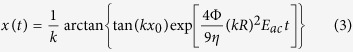


To guarantee a reliable calculation of compressibility, several parameters should be known or previously assessed with good accuracy: i) the viscosity term (*η*) was determined through the optical shooting experiments previously shown; ii) the cellular density (*ρ*_*cell*_) for both MCF7 and MDA was assumed to be 1068 kg/m^3^, as reported in the literature^45^,^51^; iii) the acoustic energy density (*E*_*ac*_) inside the microchannel was derived by analyzing the trajectories of standard polystyrene microbeads, which were mixed with cells inside the culture medium. After all these parameters are properly assessed, it is then possible to fit the acoustophoresis trajectory of each cell, thus having, by the combination of Eqs (2) and (3), the cellular compressibility as the only free parameter. In Fig. 3(c), the scatter plot of acoustic compressibility of MCF7 and MDA versus cell size is reported. Two histograms, above and on the right of the scatter diagram, are directly derived by considering only the corresponding parameter. From the cell size histogram, it is clearly shown that MCF7 and MDA cell lines have the same cell size distribution, however, the compressibility of MDA is generally higher than that of MCF7 cells.

#### Cellular optical stretching and optical deformability

After assessing the AC of the cells, we investigated their optical deformability. As the procedure for the optical stretching measurement is already well known, and can be found in details elsewhere^7^,^27^, we will just briefly recall the main numerical parameters. Low laser power (25 mW per waveguide) is applied to trap a single cell after the suspension is injected into the central microchannel. Once a single cell is trapped, the flux is stopped (to prevent multiple-cell trapping) and when the cell is in a stable equilibrium, few seconds after trapping, the measurement is started: the laser power is raised to 1 W per side for 5 s (during which the cell is stretched), and then the optical power is reduced again to 25 mW for additional 5 s, so as to allow for cell shape recovery before releasing the cell. During this 10 s process, images are acquired and saved automatically at 15 fps, to allow subsequent image processing and data analysis.

In these experiments, extra acoustophoresis is also applied to pre-focus cells to the channel centre where the pressure node is positioned. This also corresponds to the stable optical trapping point, allowing for continuous optical stretching and faster measurement as reported in literature^52^. The optical deformation is defined as the cell contour ellipticity variation as in the following equation:


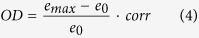


where *corr* is a correction term taking into account the different force distribution on the surface of cells of different dimensions, while *e*_0_ and *e*_*max*_ are the cell ellipticity before stretching and at the maximum stretching point^7^.

**Figure 4:**
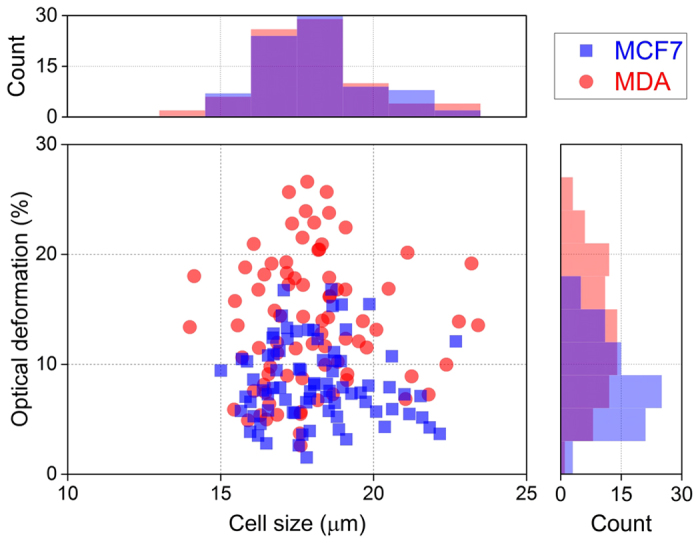
Scatter plot of optical deformation versus cell size for MCF7 and MDA cells. They show a very similar cell size, 17.9 ± 1.6 μm, but different optical deformation, 8.4 ± 3.4% for MCF7 and 13.7 ± 6.0% for MDA.

In Fig. 4, we report the cells’ OD versus the cell size. From the cell size histogram, it appears that MCF7 and MDA cell lines have the same cell size distribution, in good agreement with the observation of the cell size distribution in the AC measurement. However, the optical deformation of MDA cells covers a significantly wider range than that of MCF7 cells, especially in the high deformability (>15%) range.

It’s worth mentioning that Young’s modulus normally can be derived from the cell optical stretching measurement under the linear elastic membrane approximation for small deformation. However, in our case the cellular deformation for both MCF7 and MDA covers a very wide range, from less than 5% to more than 20%, thus making it unfeasible to accurately determine the Young’s modulus for each studied cell. For this reason, cellular optical deformability, which is a simple indicator for cellular mechanical characterization and has been successfully applied in many studies, is used as reference parameter in this study.

#### Determination of cellular acoustic compressibility and optical deformability on the same cell

**Figure 5:**
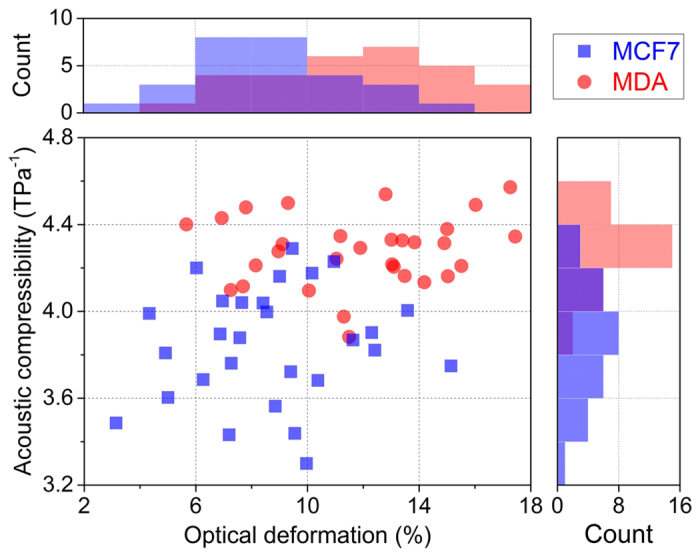
Scatter plot of cellular acoustic compressibility versus optical deformation for both MCF7 and MDA cell lines.

To further check the relationship between cellular acoustic compressibility and optical deformability, we combined both AC and OD measurements on the same cell by exploiting the two functionalities available in our chip. The scatter plot in Fig. 5 shows the acoustic compressibility versus the optical deformation for each analyzed cell. Pearson correlation coefficients between AC and OD were calculated for both MCF7 and MDA cells and turned out to be around 0.12 for both cell samples, thus meaning that no clear correlation between these two parameters is present, i.e., a higher optical deformability does not imply a higher compressibility. To evaluate the possibility to exploit these two parameters for distinguishing the two cell samples, statistic U-tests were performed separately for OD and AC on the two cell samples; the obtained p-values for both OD and AC were lower than 10^−3^. This shows that both parameters can highlight a statistically significant difference between the two samples; however this does not imply that the two parameters allows a proper cell-type identification with the same accuracy.

As it can be easily observed from Fig. 5, the overlap between two sample distributions of the AC parameter is significantly narrower than that of OD, thus suggesting that AC could be a more reliable parameter for correct cell-type identification. In order to quantitatively, and not merely qualitatively, investigate this aspect, it is possible to compare the minimum “cell-identification error” that can be obtained using AC or OD as the parameter for identification. The results obtained here indicate that if one uses an OD threshold-value of ≈11%, the cell identification based on OD introduces an error of 25%; while this error drops to 12% by using AC (threshold ≈4.07 TPa^−1^).

**Figure 6:**
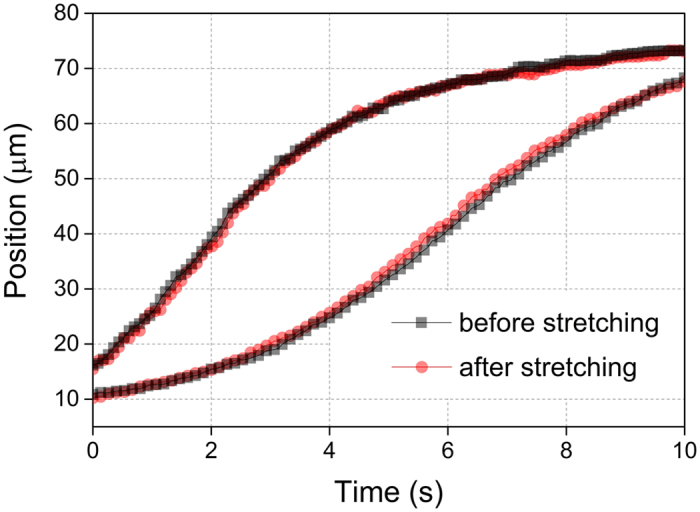
Acoustophoresis trajectory of the same two MDA cells before and after optical stretching.

Since the determination of cellular mechanical properties can give important information on cell features, devices able to study more than a single physical parameter on the same cell, like the one presented here, can be highly valuable tools, provided that the two measurements do not influence each other. For this reason, we investigated if the optical stretching procedure could modify the cell AC value, by performing two acoustophoresis measurements on the same cell before and after optical stretching. The “complementary” condition (i.e. optical stretching performed before and after AC assessment) was not investigated, as we already demonstrated in a recent study^52^, realized with the same chip, that cellular acoustophoresis does not affect the optical deformation measurement. The obtained results (two examples regarding MDA cells are shown in Fig. 6) highlight that for both MCF7 and MDA cells, optical stretching does not affect the acoustophoresis trajectory. As known, the acoustophoretic response of cells is influenced by their volume and AC. The cell volume change produced by optical stretching experiments is not significant even in the maximum-deformation configuration (OD is defined as the cell shape ellipticity variation and can be larger than 30% even with a small volume change of less than 2%). Therefore, it was not expected that cellular compressibility should be affected by optical stretching.

## Discussion

In this work, we present an integrated microfluidic chip realized by femtosecond laser micromachining technology. This chip has two distinct functions applicable for determination of the mechanical characteristics of a single cell: optical stretching for optical deformability (OD) and acoustophoresis for acoustic compressibility (AC). Additionally, even if this functionality was not introduced in the tested microchip, by using a double-Y microchannels configuration, single-cell sorting and recovery on the basis of mechanical properties can be achieved with very high efficiency. This would allow compensating for the inherent low-throughput associated with the considered characterization techniques.

Before using the device for cell-mechanics evaluations, a special effort was dedicated to the calibration of system parameters in order to obtain reliable cell measurements. The chip was then successfully exploited to measure both AC and OD of cells belonging to two human breast cancer lines: MCF7 and MDA-MB231. The obtained results clearly indicate that MDA-MB231 has both higher AC and OD than MCF7. This finding is in good agreement with recent studies^22^,^45^. The two cell lines have different metastatic potential and we speculate that the higher compressibility and deformability of MDA-MB231 can be associated with its elevated metastatic nature and can be correlated to the fact that metastatic carcinoma cells have a high invasive ability, and therefore require higher compressibility and deformability to migrate through the extra-cellular matrix and to move inside the original tissue.

After this separate analysis, combined measurements of cellular acoustic compressibility and optical deformability were directly realized on the same cell and it is found that there is no clear relationship between these two parameters in each cell sample, i.e., higher compressibility does not imply higher optical deformability. We speculate that this observation can be explained taking into account that stretching induced OD is related to an external membrane shape change and may involve contributions from cytoskeletal constituents inside the cell, while AC is a physical parameter mainly related to cellular liquid components, like the cytosol. In order to point out the differences between the two parameters, it may be helpful to consider two “extreme” cases, and the consequences on AC and OD. If we consider a fixed-volume (and variable surface) object, e.g. a plastic balloon filled with a non-compressible fluid, we have an object with zero AC, but large OD. On the other side, if we consider as an example a cube with variable-length edges, but non-deformable corners, then we could have a high AC, but no detectable change of the “shape” using the OD parameter.

Furthermore, we checked if the two measurement techniques influence the result of the other method when the parameters are measured using the device investigated. In earlier work^52^, we demonstrated that acoustophoresis applied to the cells does not affect their optical deformability. In the experiments reported here, the influence of optical stretching of the cells on their acoustic compressibility was considered, and the results show that the cellular acoustic compressibility after optical stretching was the same as before. Therefore, it is demonstrated that the two functions in our chip do not affect each other when performed on the same cell, thus allowing the simultaneous determination of two important mechanical parameters on single cells.

In conclusion, the chip presented here, together with the proposed measurement protocols, constitute a step forward in the characterization of cellular mechanical properties and thus in the possible identification of cells with specific biological properties.

## Materials and Methods

In this section we first discuss the samples used for the experiments and then present the microchip fabrication process, and the different building blocks of the experimental apparatus. The direct combination of OD and AC measurements is realized by using a piezo-transducer attached to the optofluidic microchip to excite ultrasonic waves in both the chip and the fluid inside the microchannel^52^,^53^. All the measurements were performed at constant room temperature (22 ± 1 °C).

### Sample preparation

Two different kinds of samples were used for the measurements: polystyrene microbeads (Sigma-Aldrich) and cancer cells. The two considered cancer cell lines were characterized by a different metastatic potential: non-metastatic human breast carcinoma cells, MCF7, and their metastatic counterpart MDA-MB231. Cells were cultured in 10 cm Petri dishes in Dulbecco’s Modified Eagle’s Medium (DMEM) supplemented with 10% fetal bovine serum, penicillin (0.1/mg) and streptomycin (100 U/ml), 0.2 mM glutamine and 1× non-essential amino acid; all the listed substances were purchased from Euroclone. Cells were maintained in an incubator at 37 °C in a humidified atmosphere at 5% CO_2_. When requested for the experiments, cells were detached by adding trypsin and then suspended in culture medium without serum at a density of about 200 cells per μL, to ensure a regular presence of cells within the flow.

### Microchip fabrication

A commercially available microfluidic chip, based on a three layer technology by Translume Inc. (size 50.8 × 17.6 × 1.1 mm − L × W × H), was used as the basis of our experiments: it has a single 150 μm-wide microchannel, created in the 150 μm-thick middle layer of fused silica glass through fs-laser irradiation followed by chemical etching^54^,^55^. This layer is then sandwiched on both sides and sealed by thermal bonding with two 500 μm-thick fused-silica polished glass slides. The top one has two through-holes aligned with the slot terminals so as to form the top access of the embedded microchannel. The reservoirs are glued with two Luer connectors allowing easy connection between the microchip internal fluidic part and the external fluidic circuit. Thanks to this technology, the central channel has an almost ideal square cross section of 150 × 150 μm^2^, with high optical quality of all the walls, which can provide a high resolution imaging of the flowing sample.

**Figure 7:**
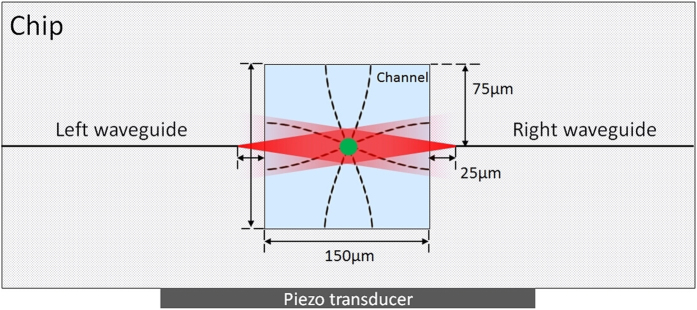
Illustration of the chip cross section. Dashed lines inside the channel represent the amplitude of the resonant pressure waves both in the horizontal and in the vertical directions.

Optical waveguides for cellular optical stretching were integrated in the middle glass layer by fs-laser writing, allowing for an almost perfect alignment between the two facing waveguides^56^. Differently from a conventional optical stretcher, where the optical waveguides or fibers are usually positioned in the lower half of the microchannel so that the beam may intercept a high quantity of cells, in this device the optical waveguides were realized exactly at half the microchannel height, 75 μm above the microchannel floor. This design choice is related to the fact that acoustophoretic prefocusing is planned to be applied to the chip^52^, making cell acquisition easier and leading to faster cell analysis. The cross-section of the finished chip is illustrated in Fig. 7, where the resonant acoustic wave is schematically indicated by dashed lines. The waveguides end-faces are separated by 200 μm, so that the beams experience free-space propagation first in the glass (for 25 μm) and then in the microchannel. As a final fabrication step, the chip lateral surfaces were polished to optical quality and two optical fibers (Corning Hi1060) were pigtailed to the chip to guarantee stable, and low-loss, fiber-to-waveguide beam coupling.

### Experimental setup and procedure

**Figure 8:**
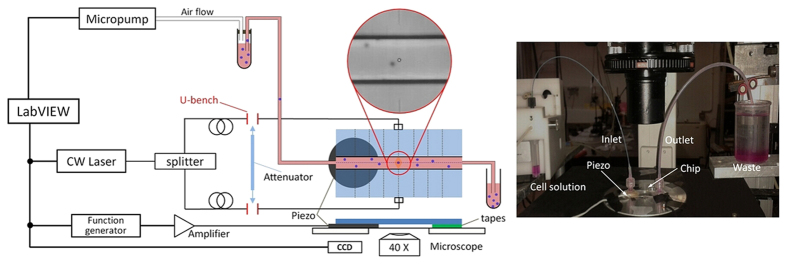
Schematic diagram of the experiment setup and an image of the microchip mounted on a phase contrast microscope. Local enlarged image in the left panel shows a single bead trapped in the middle of the microchannel by optical force from the two facing waveguides.

A sketch of the experimental setup is shown in Fig. 8. The whole system can be divided into four main parts: fluidic, optical, acoustic and image acquisition. All controls are realized through a custom made Labview program. Regarding the fluidic circulation, the central channel of our microchip was connected to two external tubings: one was used to inject the bead or cell sample by means of a high precision micropump (MFCSTM-EZ), and the other one was kept immersed in water as a waste disposal to prevent any turbulence or pressure fluctuation caused by droplet variation over the whole experiment duration.

In the optical path, a CW Yb-doped fiber laser (YLD-10-1064, IPG Photonics, PMAX = 10 W at 1070 nm) was employed as light source and its optical power was evenly split into two single mode fibers, which are separately spliced to two fiber-to-fiber U-benches, and then pigtailed to the optical waveguides integrated inside the chip. The U-benches allow us to easily access the optical beam or manipulate its power, e.g. to reduce the optical power in one waveguide by simply putting an optical attenuator inside one U-bench when a trapping position displacement is needed. We also took advantage of the U-benches to obtain a collimated laser beam for the refractive index measurements as discussed in the results section.

For the acoustic actuation part, a piezo ceramic driven by an external amplified function generator was attached beneath the microchip by using glycerol in-between to increase the acoustic wave coupling. It should be noted that the piezo was chosen to have an eigen-frequency of 5 MHz, which roughly corresponds to the calculated frequency of the single-node acoustic resonance of the microchannel^52^. Finally, about the imaging acquisition part, the chip was mounted on an inverted phase contrast microscope, equipped with a 40× objective and connected to a CCD.

**Figure 9:**
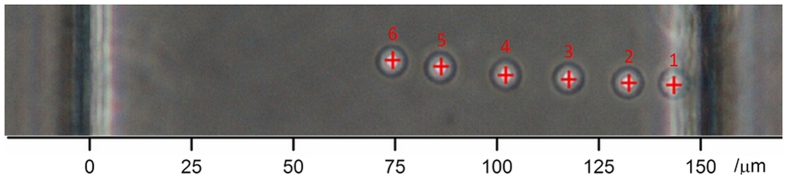
Microbead-tracking example. The shown image is obtained by overlapping 6 frames, recorded every 2 s during the microbead acoustophoresis measurement. The small but progressive shift in the vertical position of the bead is related to the flux fluctuation inside the microchannel.

The high-quality imaging allowed by the used microchip is fundamental to allow a precise determination of both OD and AC, which requires an accurate analysis of the images obtained from the experiments. In order to assess the size of trapped cells, a customized edge-detection algorithm for cellular contour recognition is applied to each image saved during optical stretching measurements, thus allowing us to extract the OD of each measured cell with subpixel accuracy^7^. On the other side, in order to study the movement produced by acoustophoresis across the microchannel of the polystyrene microbeads or cells, a free video analysis software *Tracker 4.87* was used to identify in each frame the center position of the studied bead or cell. In Fig. 9, an example of microbead-movement tracking is shown.

## Additional Information

**How to cite this article**: Yang, T. *et al.* A comprehensive strategy for the analysis of acoustic compressibility and optical deformability on single cells. *Sci. Rep.* **6**, 23946; doi: 10.1038/srep23946 (2016).
