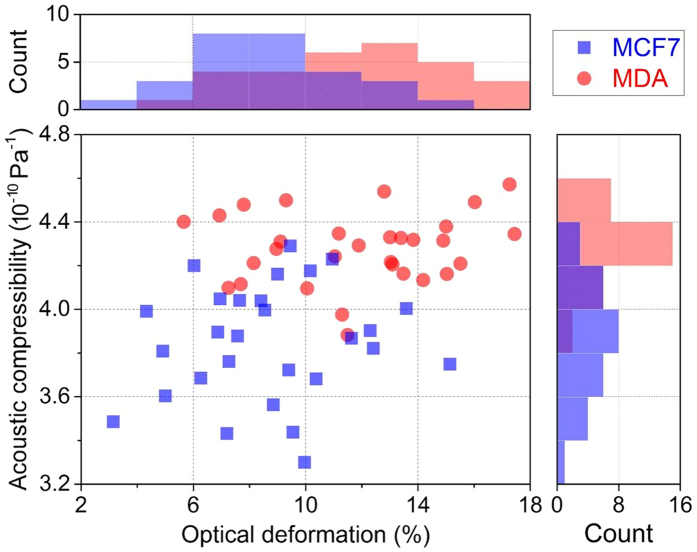# Corrigendum: A comprehensive strategy for the analysis of acoustic compressibility and optical deformability on single cells

**DOI:** 10.1038/srep40974

**Published:** 2017-01-19

**Authors:** Tie Yang, Francesca Bragheri, Giovanni Nava, Ilaria Chiodi, Chiara Mondello, Roberto Osellame, Kirstine Berg-Sørensen, Ilaria Cristiani, Paolo Minzioni

Scientific Reports 6: Article number: 23946; 10.1038/srep23946 published online: 04 04 2016; updated: 01 19 2017.

This Article contains errors. In Figure 3c, the y-axis ‘Acoustic compressibility (10^−10^ Pa^−1^)’ is incorrectly given as ‘Acoustic compressibility (TPa^−1^)’. The correct Figure 3c appears below as Figure 1.

The legend of Figure 3 contains errors.

‘Cellular acoustic compressibility versus cell size: MCF7 and MDA show a very similar cell size, 17.3 ± 1.0 μm, but different acoustic compressibility, 3.8 ± 0.3 TPa^−1^ for MCF7 and 4.3 ± 0.2  TPa^−1^ for MDA’.

should read:

‘Cellular acoustic compressibility versus cell size: MCF7 and MDA show a very similar cell size, 17.3 ± 1.0 μm, but different acoustic compressibility, 3.8 ± 0.3 10^−10^ Pa^−1^ for MCF7 and 4.3 ± 0.2 10^−10^ Pa^−1^ for MDA’.

In Figure 5, the y-axis ‘Acoustic compressibility (10^−10^ Pa^−1^)’ is incorrectly given as ‘Acoustic compressibility (TPa^−1^)’. The correct Figure 5 appears below as Figure 2. The figure legend is correct.

In the Results section under the subheading ‘Determination of cellular acoustic compressibility and optical deformability on the same cell’,

“The results obtained here indicate that if one uses an OD threshold-value of ≈11%, the cell identification based on OD introduces an error of 25%; while this error drops to 12% by using AC (threshold ≈ 4.07 TPa^−1^)”.

should read:

“The results obtained here indicate that if one uses an OD threshold-value of ≈11%, the cell identification based on OD introduces an error of 25%; while this error drops to 12% by using AC (threshold ≈ 4.07 10^−10^ Pa^−1^).”

## Figures and Tables

**Figure 1:**
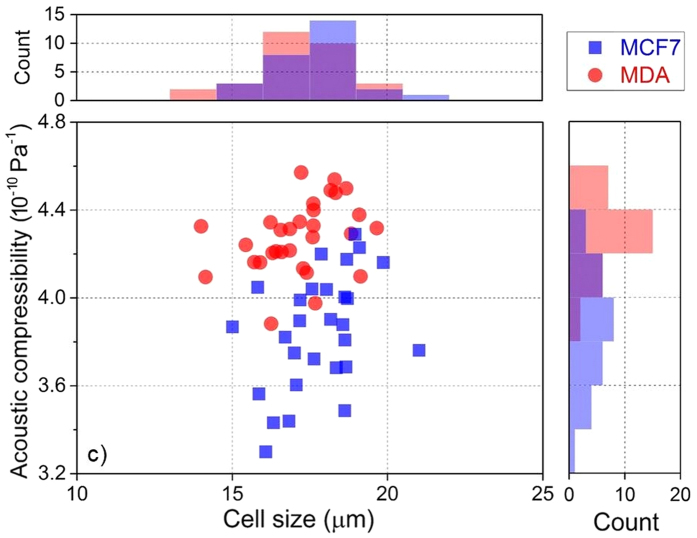


**Figure 2:**